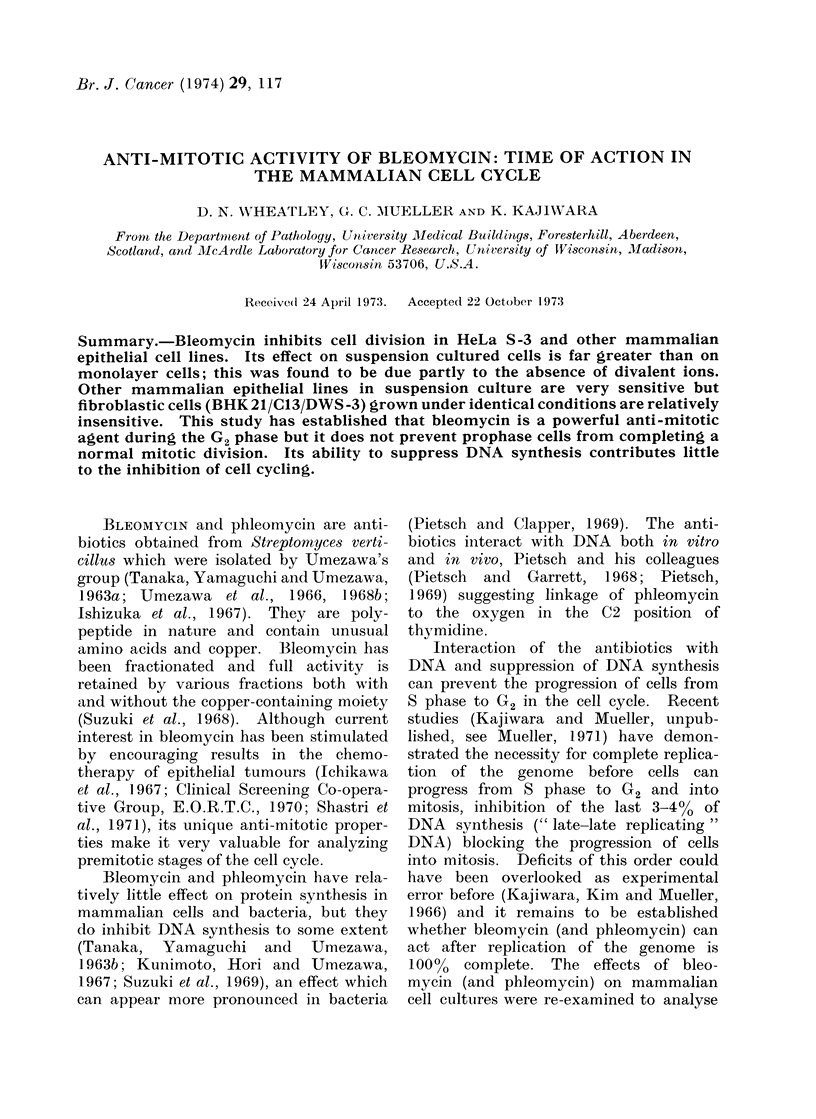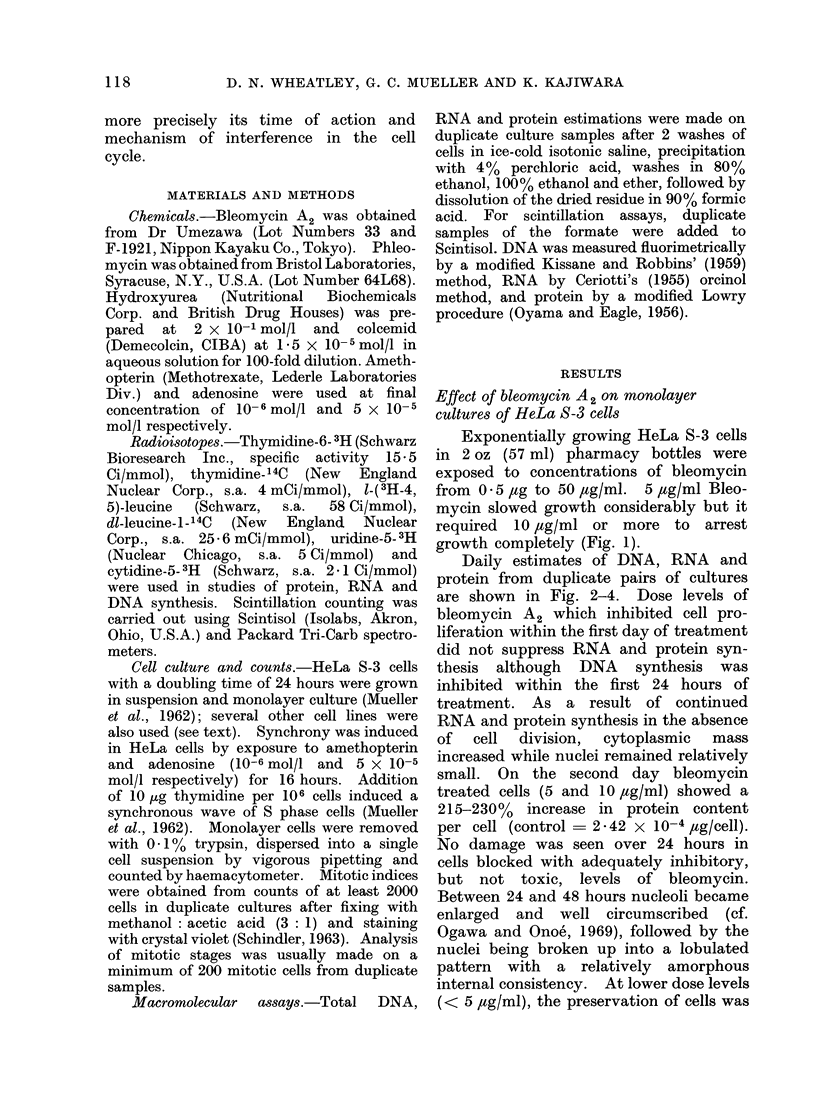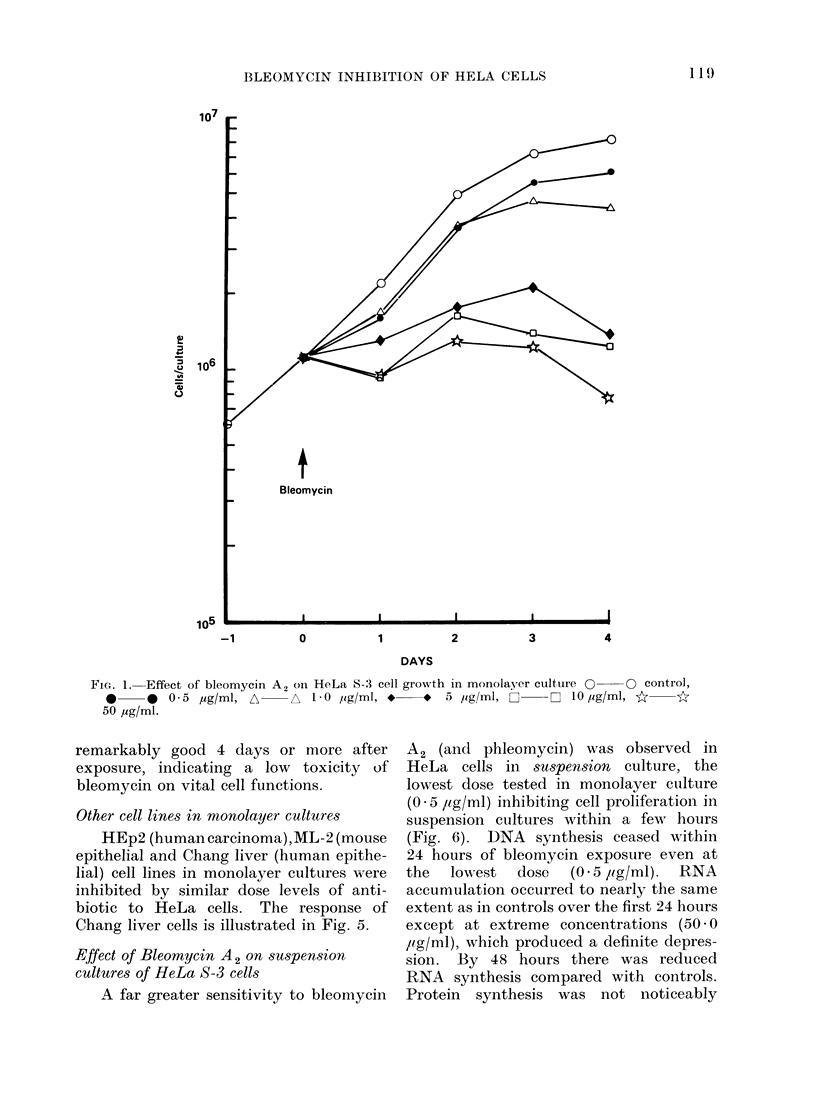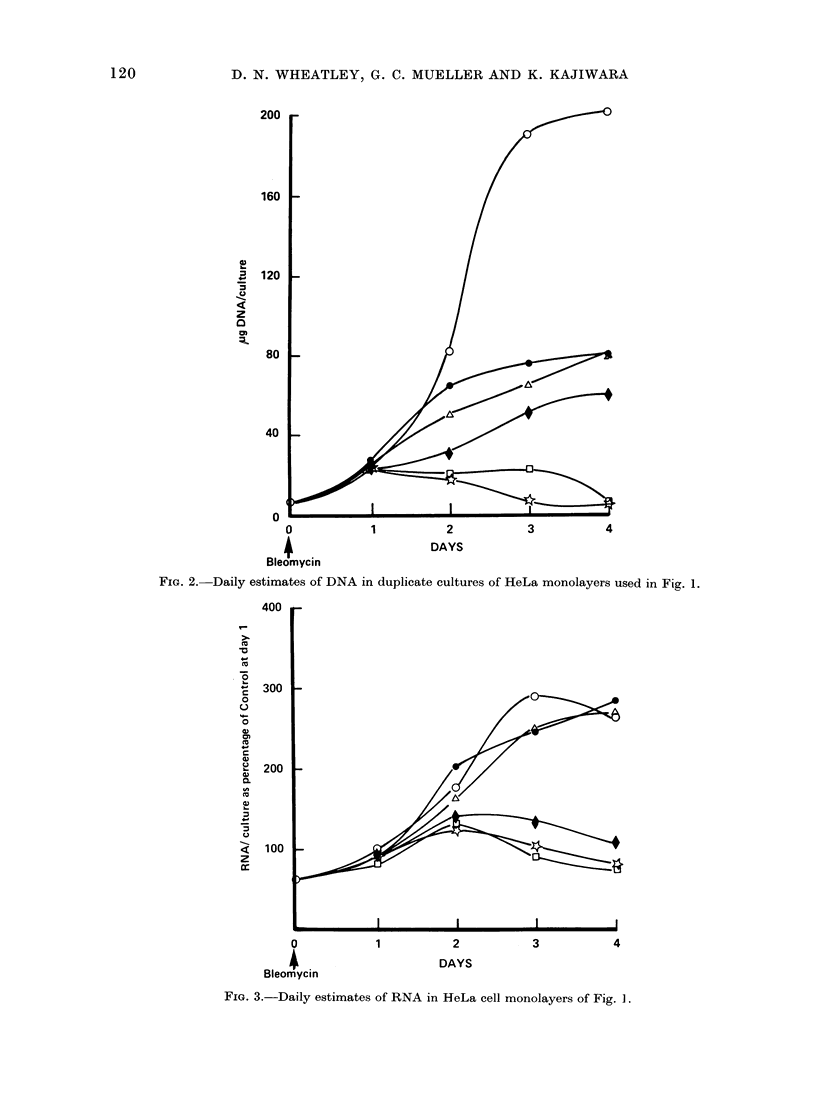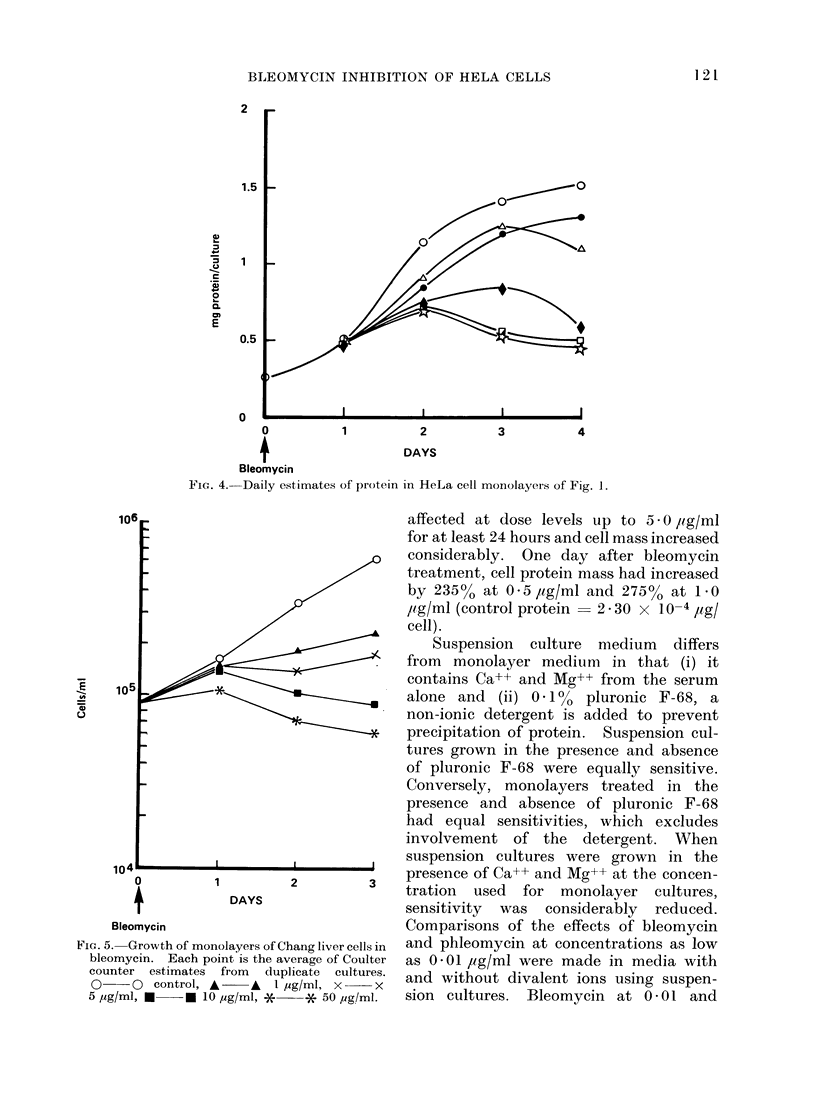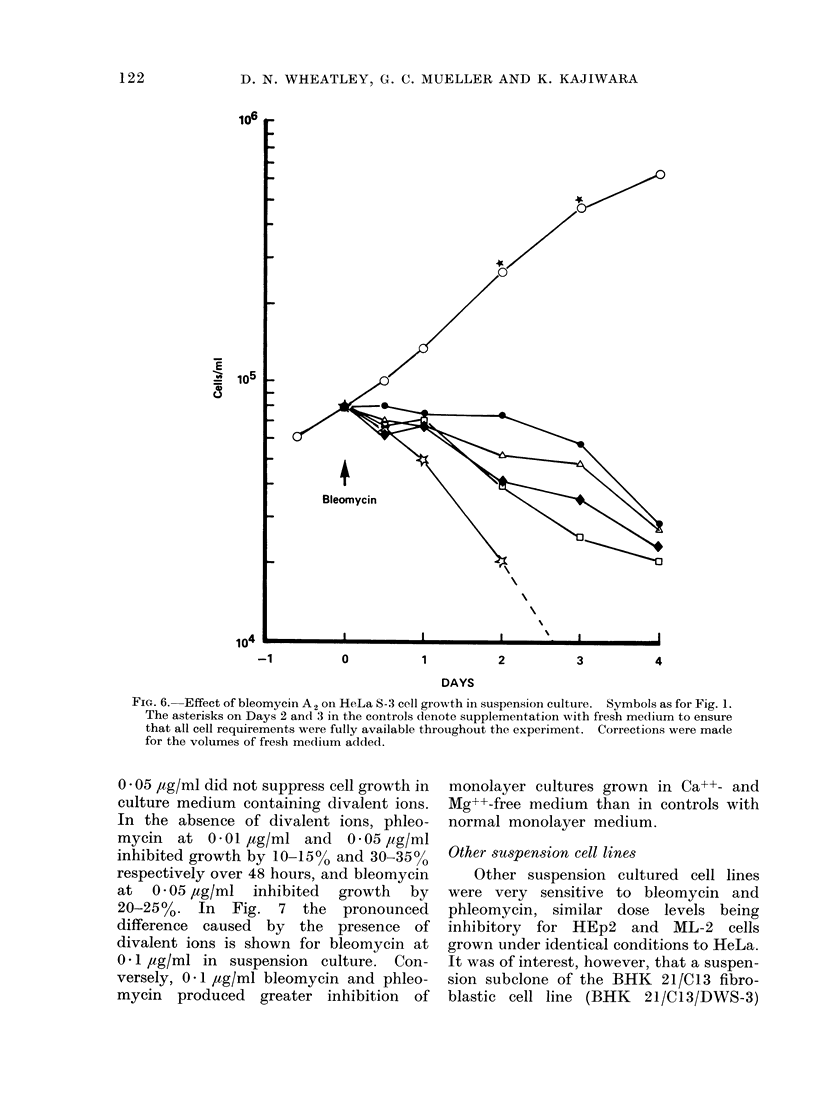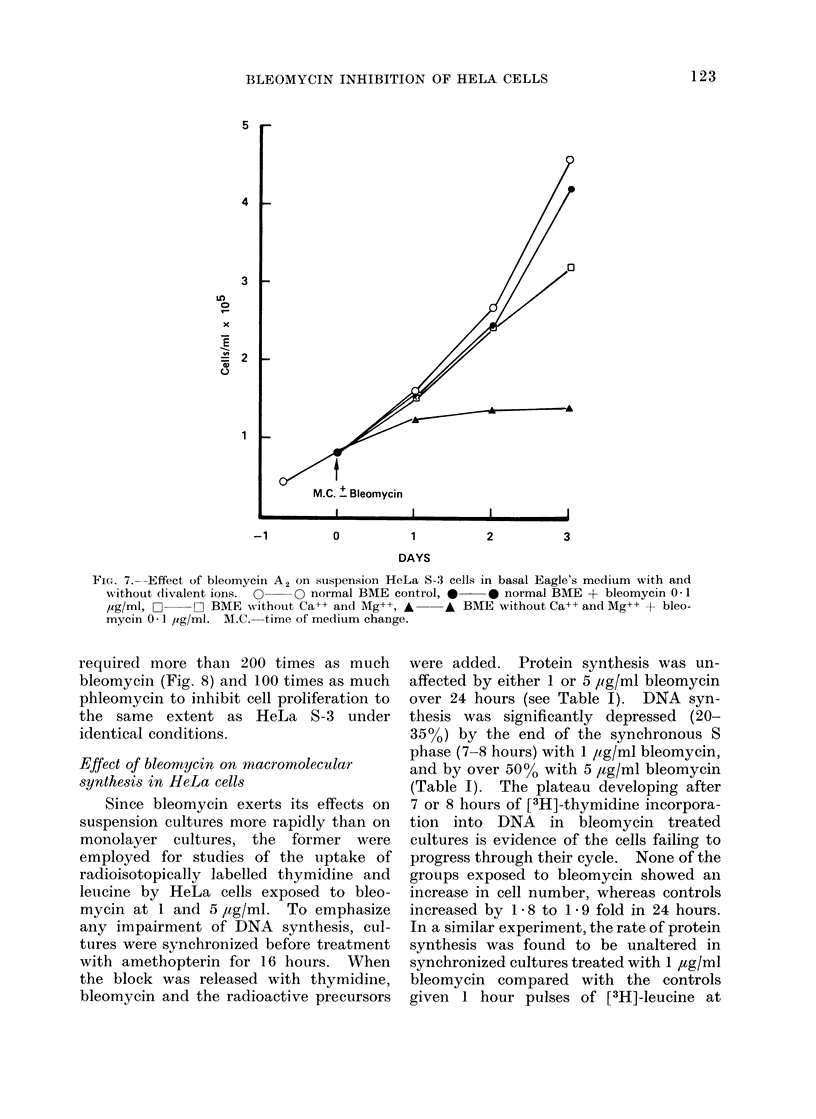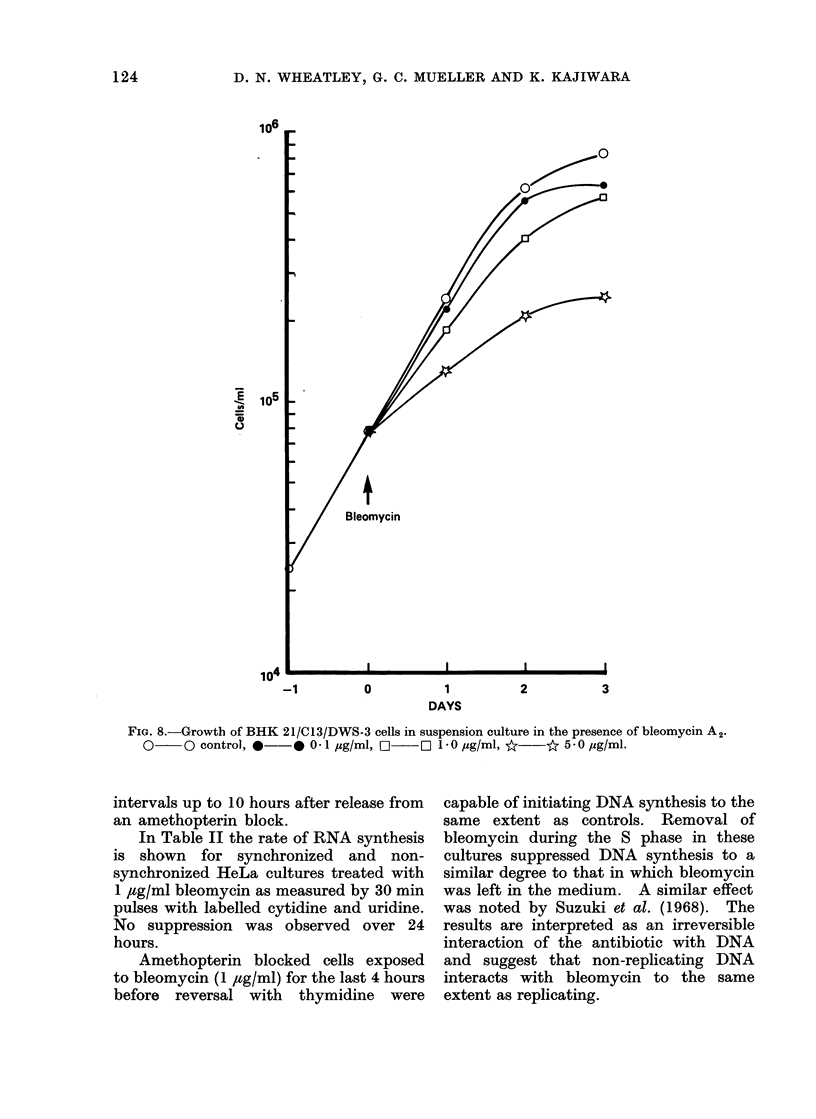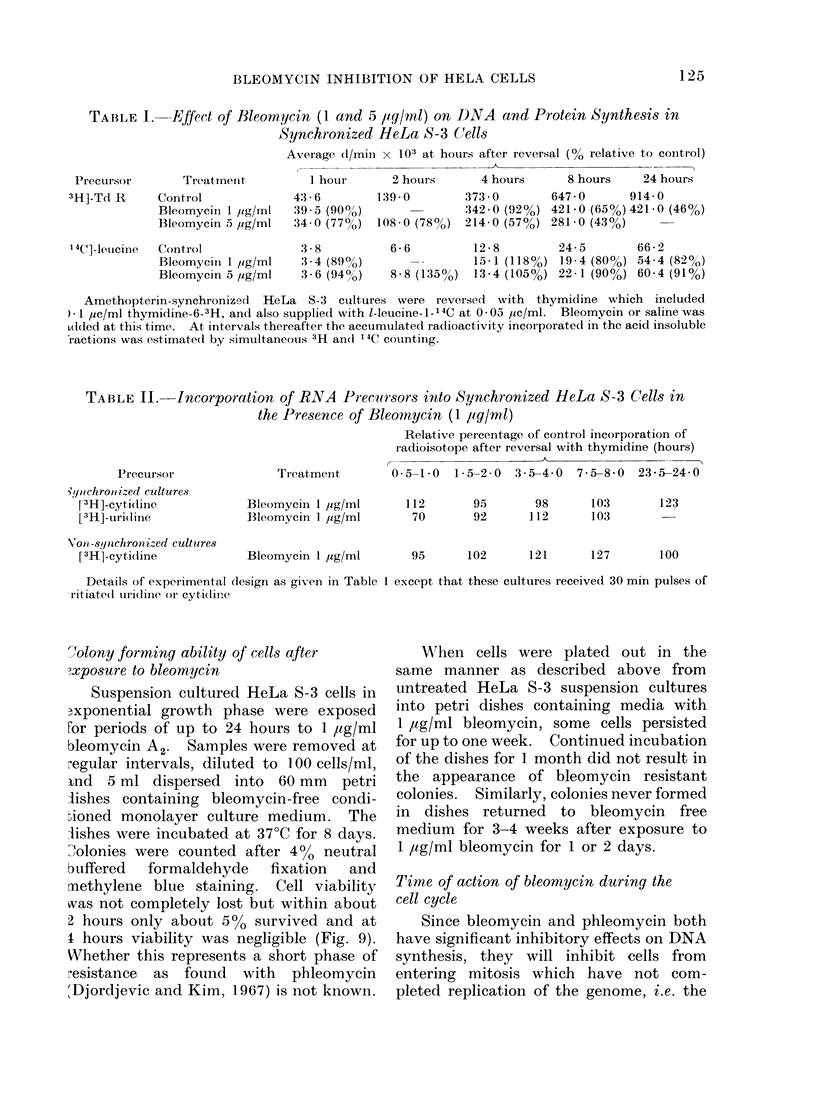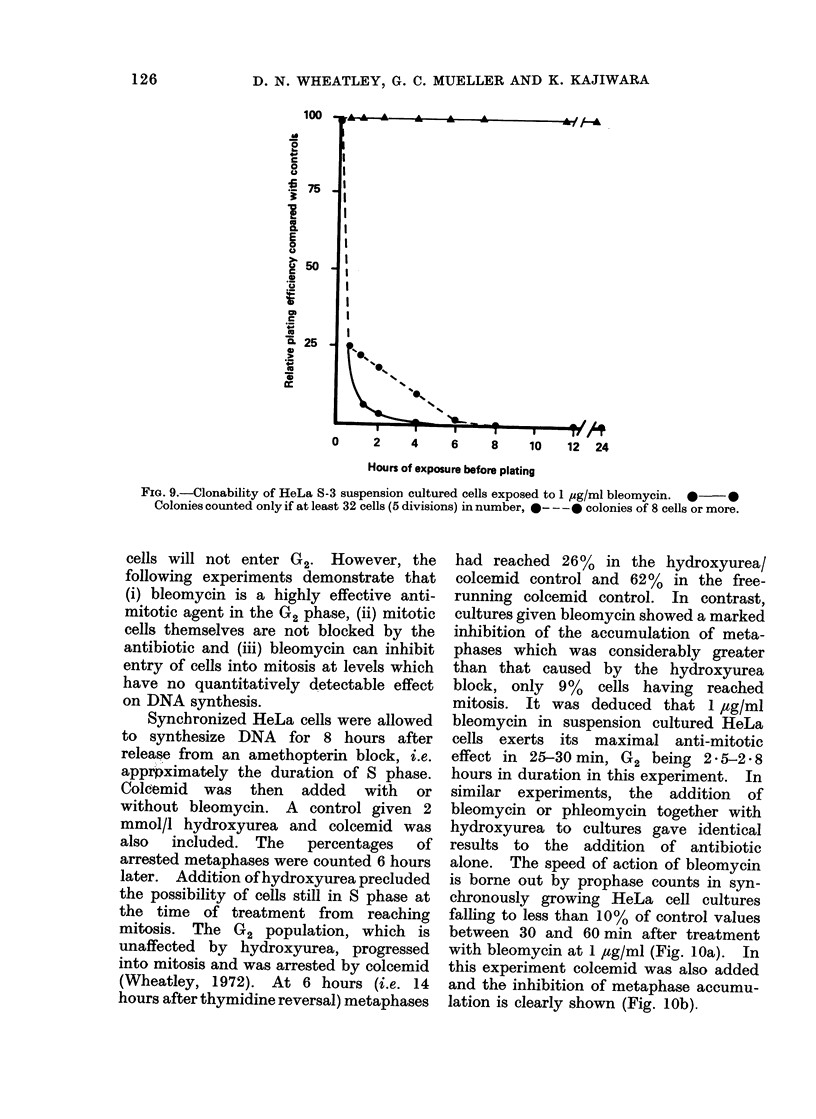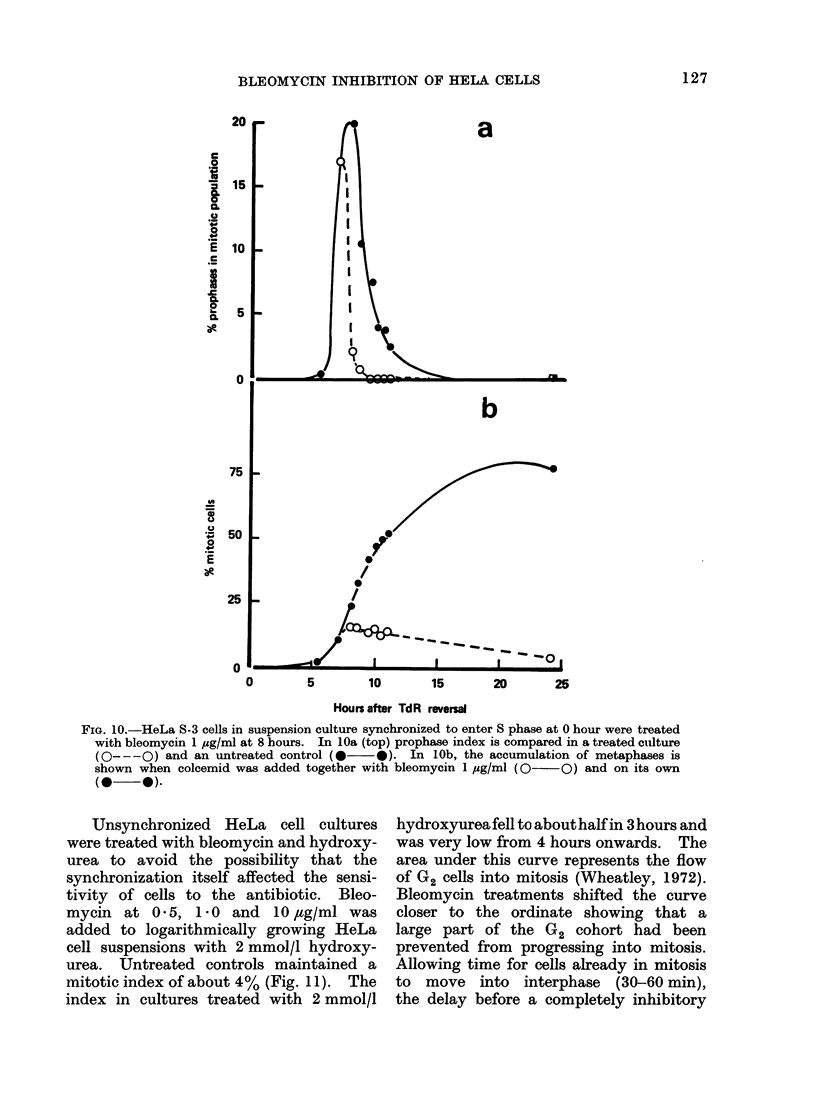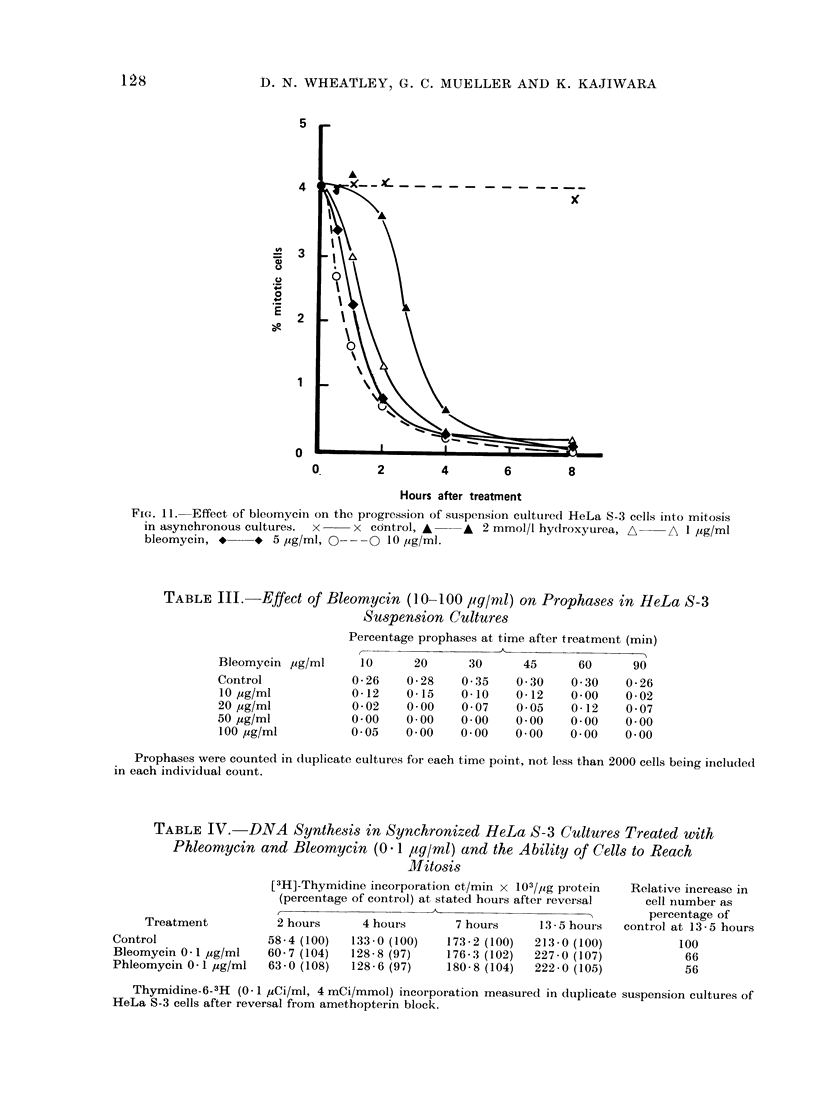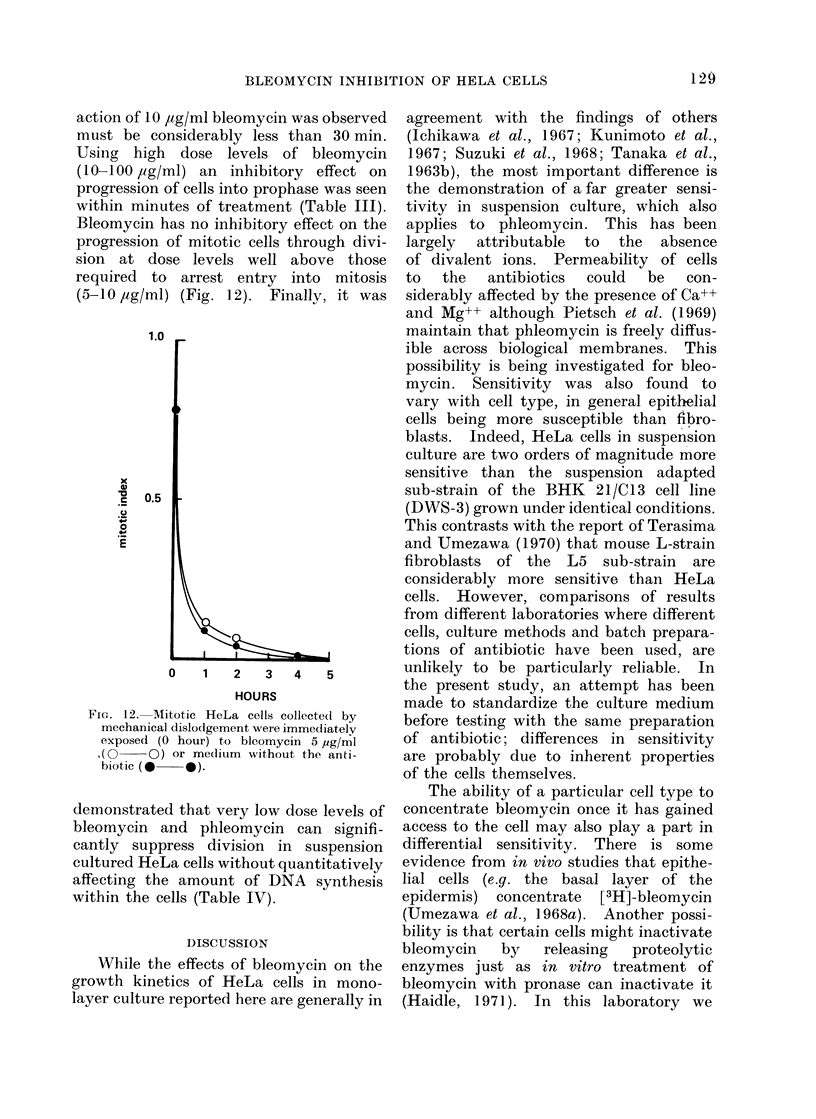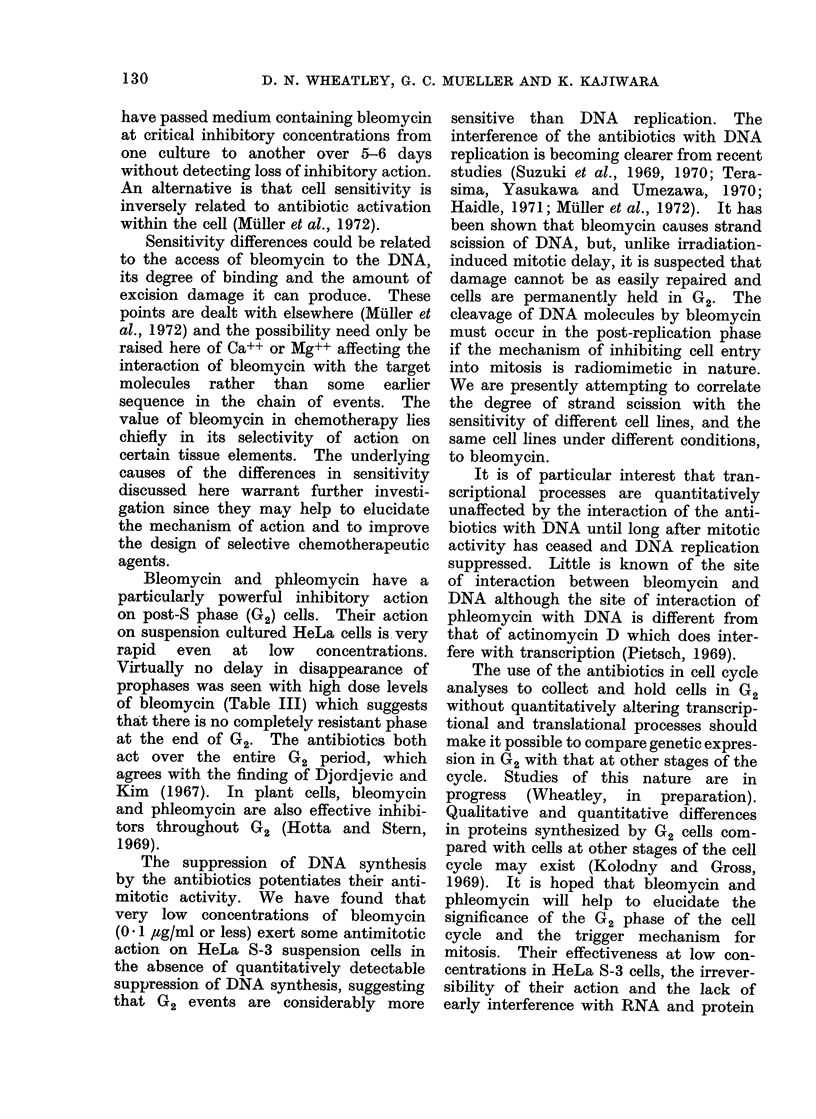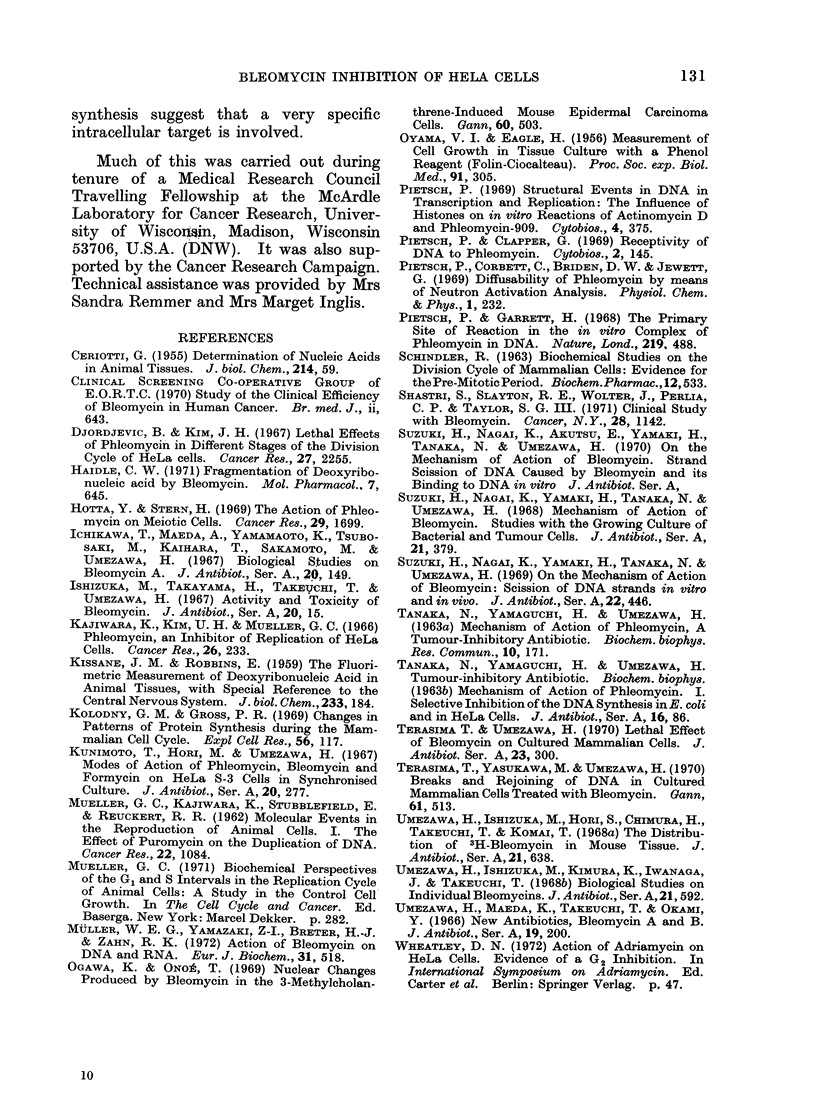# Anti-mitotic Activity of Bleomycin: Time of Action in the Mammalian Cell Cycle

**DOI:** 10.1038/bjc.1974.48

**Published:** 1974-02

**Authors:** D. N. Wheatley, G. C. Mueller, K. Kajiwara

## Abstract

Bleomycin inhibits cell division in HeLa S-3 and other mammalian epithelial cell lines. Its effect on suspension cultured cells is far greater than on monolayer cells; this was found to be due partly to the absence of divalent ions. Other mammalian epithelial lines in suspension culture are very sensitive but fibroblastic cells (BHK 21/C13/DWS-3) grown under identical conditions are relatively insensitive. This study has established that bleomycin is a powerful anti-mitotic agent during the G_2_ phase but it does not prevent prophase cells from completing a normal mitotic division. Its ability to suppress DNA synthesis contributes little to the inhibition of cell cycling.


*Br. J. Cancer* (1974) **29**, 117

### ANTI-MITOTIC ACTIVITY OF BLEOMYCIN: TIME OF ACTION IN THE MAMMALIAN CELL CYCLE

D. N. WHEATLEY, G. C. MUELLER AND K. KAJIWARA

*From the Department of Pathology, University Medical Buildings, Foresterhill, Aberdeen, Scotland, and McArdle Laboratory for Cancer Research, University of Wisconsin, Madison, Wisconsin* 53706, *U.S.A.*

Received 24 April 1973. Accepted 22 October 1973

**Summary.—Bleomycin inhibits cell division in HeLa S-3 and other mammalian epithelial cell lines. Its effect on suspension cultured cells is far greater than on monolayer cells; this was found to be due partly to the absence of divalent ions. Other mammalian epithelial lines in suspension culture are very sensitive but fibroblastic cells (BHK21/C13/DWS-3) grown under identical conditions are relatively insensitive. This study has established that bleomycin is a powerful anti-mitotic agent during the $G_2$ phase but it does not prevent prophase cells from completing a normal mitotic division. Its ability to suppress DNA synthesis contributes little to the inhibition of cell cycling.**

BLEOMYCIN and phleomycin are antibiotics obtained from *Streptomyces verticillus* which were isolated by Umezawa's group (Tanaka, Yamaguchi and Umezawa, 1963*a*; Umezawa *et al.*, 1966, 1968*b*; Ishizuka *et al.*, 1967). They are polypeptide in nature and contain unusual amino acids and copper. Bleomycin has been fractionated and full activity is retained by various fractions both with and without the copper-containing moiety (Suzuki *et al.*, 1968). Although current interest in bleomycin has been stimulated by encouraging results in the chemotherapy of epithelial tumours (Ichikawa *et al.*, 1967; Clinical Screening Co-operative Group, E.O.R.T.C., 1970; Shastri *et al.*, 1971), its unique anti-mitotic properties make it very valuable for analyzing premitotic stages of the cell cycle.

Bleomycin and phleomycin have relatively little effect on protein synthesis in mammalian cells and bacteria, but they do inhibit DNA synthesis to some extent (Tanaka, Yamaguchi and Umezawa, 1963*b*; Kunimoto, Hori and Umezawa, 1967; Suzuki *et al.*, 1969), an effect which can appear more pronounced in bacteria (Pietsch and Clapper, 1969). The antibiotics interact with DNA both *in vitro* and *in vivo*, Pietsch and his colleagues (Pietsch and Garrett, 1968; Pietsch, 1969) suggesting linkage of phleomycin to the oxygen in the C2 position of thymidine.

Interaction of the antibiotics with DNA and suppression of DNA synthesis can prevent the progression of cells from S phase to $G_2$ in the cell cycle. Recent studies (Kajiwara and Mueller, unpublished, see Mueller, 1971) have demonstrated the necessity for complete replication of the genome before cells can progress from S phase to $G_2$ and into mitosis, inhibition of the last 3–4% of DNA synthesis ("late–late replicating" DNA) blocking the progression of cells into mitosis. Deficits of this order could have been overlooked as experimental error before (Kajiwara, Kim and Mueller, 1966) and it remains to be established whether bleomycin (and phleomycin) can act after replication of the genome is 100% complete. The effects of bleomycin (and phleomycin) on mammalian cell cultures were re-examined to analyse more precisely its time of action and mechanism of interference in the cell cycle.

### MATERIALS AND METHODS

*Chemicals.*—Bleomycin $A_2$ was obtained from Dr Umezawa (Lot Numbers 33 and F-1921, Nippon Kayaku Co., Tokyo). Phleomycin was obtained from Bristol Laboratories, Syracuse, N.Y., U.S.A. (Lot Number 64L68). Hydroxyurea (Nutritional Biochemicals Corp. and British Drug Houses) was prepared at $2 \times 10^{-1}$ mol/l and colcemid (Demecolcin, CIBA) at $1 \cdot 5 \times 10^{-5}$ mol/l in aqueous solution for 100-fold dilution. Amethopterin (Methotrexate, Lederle Laboratories Div.) and adenosine were used at final concentration of $10^{-6}$ mol/l and $5 \times 10^{-5}$ mol/l respectively.

*Radioisotopes.*—Thymidine-6-$^3$H (Schwarz Bioresearch Inc., specific activity 15·5 Ci/mmol), thymidine-$^{14}$C (New England Nuclear Corp., s.a. 4 mCi/mmol), *l*-($^3$H-4, 5)-leucine (Schwarz, s.a. 58 Ci/mmol), *dl*-leucine-1-$^{14}$C (New England Nuclear Corp., s.a. 25·6 mCi/mmol), uridine-5-$^3$H (Nuclear Chicago, s.a. 5 Ci/mmol) and cytidine-5-$^3$H (Schwarz, s.a. 2·1 Ci/mmol) were used in studies of protein, RNA and DNA synthesis. Scintillation counting was carried out using Scintisol (Isolabs, Akron, Ohio, U.S.A.) and Packard Tri-Carb spectrometers.

*Cell culture and counts.*—HeLa S-3 cells with a doubling time of 24 hours were grown in suspension and monolayer culture (Mueller *et al.*, 1962); several other cell lines were also used (see text). Synchrony was induced in HeLa cells by exposure to amethopterin and adenosine ($10^{-6}$ mol/l and $5 \times 10^{-5}$ mol/l respectively) for 16 hours. Addition of 10 $\mu$g thymidine per $10^6$ cells induced a synchronous wave of S phase cells (Mueller *et al.*, 1962). Monolayer cells were removed with 0·1% trypsin, dispersed into a single cell suspension by vigorous pipetting and counted by haemacytometer. Mitotic indices were obtained from counts of at least 2000 cells in duplicate cultures after fixing with methanol : acetic acid (3 : 1) and staining with crystal violet (Schindler, 1963). Analysis of mitotic stages was usually made on a minimum of 200 mitotic cells from duplicate samples.

*Macromolecular assays.*—Total DNA, RNA and protein estimations were made on duplicate culture samples after 2 washes of cells in ice-cold isotonic saline, precipitation with 4% perchloric acid, washes in 80% ethanol, 100% ethanol and ether, followed by dissolution of the dried residue in 90% formic acid. For scintillation assays, duplicate samples of the formate were added to Scintisol. DNA was measured fluorimetrically by a modified Kissane and Robbins' (1959) method, RNA by Ceriotti's (1955) orcinol method, and protein by a modified Lowry procedure (Oyama and Eagle, 1956).

### RESULTS

### *Effect of bleomycin $A_2$ on monolayer cultures of HeLa S-3 cells*

Exponentially growing HeLa S-3 cells in 2 oz (57 ml) pharmacy bottles were exposed to concentrations of bleomycin from 0·5 $\mu$g to 50 $\mu$g/ml. 5 $\mu$g/ml Bleomycin slowed growth considerably but it required 10 $\mu$g/ml or more to arrest growth completely (Fig. 1).

Daily estimates of DNA, RNA and protein from duplicate pairs of cultures are shown in Fig. 2–4. Dose levels of bleomycin $A_2$ which inhibited cell proliferation within the first day of treatment did not suppress RNA and protein synthesis although DNA synthesis was inhibited within the first 24 hours of treatment. As a result of continued RNA and protein synthesis in the absence of cell division, cytoplasmic mass increased while nuclei remained relatively small. On the second day bleomycin treated cells (5 and 10 $\mu$g/ml) showed a 215–230% increase in protein content per cell (control = $2 \cdot 42 \times 10^{-4}$ $\mu$g/cell). No damage was seen over 24 hours in cells blocked with adequately inhibitory, but not toxic, levels of bleomycin. Between 24 and 48 hours nucleoli became enlarged and well circumscribed (cf. Ogawa and Onoé, 1969), followed by the nuclei being broken up into a lobulated pattern with a relatively amorphous internal consistency. At lower dose levels ($< 5$ $\mu$g/ml), the preservation of cells was

FIG. 1.—Effect of bleomycin $A_2$ on HeLa S-3 cell growth in monolayer culture ○——○ control, ●——● 0·5 μg/ml, △——△ 1·0 μg/ml, ◆——◆ 5 μg/ml, □——□ 10 μg/ml, ☆——☆ 50 μg/ml.

remarkably good 4 days or more after exposure, indicating a low toxicity of bleomycin on vital cell functions.

*Other cell lines in monolayer cultures*

HEp2 (human carcinoma), ML-2 (mouse epithelial and Chang liver (human epithelial) cell lines in monolayer cultures were inhibited by similar dose levels of antibiotic to HeLa cells. The response of Chang liver cells is illustrated in Fig. 5.

*Effect of Bleomycin $A_2$ on suspension cultures of HeLa S-3 cells*

A far greater sensitivity to bleomycin $A_2$ (and phleomycin) was observed in HeLa cells in *suspension* culture, the lowest dose tested in monolayer culture (0·5 μg/ml) inhibiting cell proliferation in suspension cultures within a few hours (Fig. 6). DNA synthesis ceased within 24 hours of bleomycin exposure even at the lowest dose (0·5 μg/ml). RNA accumulation occurred to nearly the same extent as in controls over the first 24 hours except at extreme concentrations (50·0 μg/ml), which produced a definite depression. By 48 hours there was reduced RNA synthesis compared with controls. Protein synthesis was not noticeably

FIG. 2.—Daily estimates of DNA in duplicate cultures of HeLa monolayers used in Fig. 1.

FIG. 3.—Daily estimates of RNA in HeLa cell monolayers of Fig. 1.

FIG. 4.—Daily estimates of protein in HeLa cell monolayers of Fig. 1.

FIG. 5.—Growth of monolayers of Chang liver cells in bleomycin. Each point is the average of Coulter counter estimates from duplicate cultures. ○——○ control, ▲——▲ 1 μg/ml, ×——× 5 μg/ml, ■——■ 10 μg/ml, ✳——✳ 50 μg/ml.

affected at dose levels up to 5·0 μg/ml for at least 24 hours and cell mass increased considerably. One day after bleomycin treatment, cell protein mass had increased by 235% at 0·5 μg/ml and 275% at 1·0 μg/ml (control protein = $2 \cdot 30 \times 10^{-4}$ μg/cell).

Suspension culture medium differs from monolayer medium in that (i) it contains $Ca^{++}$ and $Mg^{++}$ from the serum alone and (ii) 0·1% pluronic F-68, a non-ionic detergent is added to prevent precipitation of protein. Suspension cultures grown in the presence and absence of pluronic F-68 were equally sensitive. Conversely, monolayers treated in the presence and absence of pluronic F-68 had equal sensitivities, which excludes involvement of the detergent. When suspension cultures were grown in the presence of $Ca^{++}$ and $Mg^{++}$ at the concentration used for monolayer cultures, sensitivity was considerably reduced. Comparisons of the effects of bleomycin and phleomycin at concentrations as low as 0·01 μg/ml were made in media with and without divalent ions using suspension cultures. Bleomycin at 0·01 and

Fig. 6.—Effect of bleomycin $A_2$ on HeLa S-3 cell growth in suspension culture. Symbols as for Fig. 1. The asterisks on Days 2 and 3 in the controls denote supplementation with fresh medium to ensure that all cell requirements were fully available throughout the experiment. Corrections were made for the volumes of fresh medium added.

0·05 $\mu$g/ml did not suppress cell growth in culture medium containing divalent ions. In the absence of divalent ions, phleomycin at 0·01 $\mu$g/ml and 0·05 $\mu$g/ml inhibited growth by 10–15% and 30–35% respectively over 48 hours, and bleomycin at 0·05 $\mu$g/ml inhibited growth by 20–25%. In Fig. 7 the pronounced difference caused by the presence of divalent ions is shown for bleomycin at 0·1 $\mu$g/ml in suspension culture. Conversely, 0·1 $\mu$g/ml bleomycin and phleomycin produced greater inhibition of monolayer cultures grown in $Ca^{++}$- and $Mg^{++}$-free medium than in controls with normal monolayer medium.

*Other suspension cell lines*

Other suspension cultured cell lines were very sensitive to bleomycin and phleomycin, similar dose levels being inhibitory for HEp2 and ML-2 cells grown under identical conditions to HeLa. It was of interest, however, that a suspension subclone of the BHK 21/C13 fibroblastic cell line (BHK 21/C13/DWS-3)

FIG. 7.—Effect of bleomycin $A_2$ on suspension HeLa S-3 cells in basal Eagle's medium with and without divalent ions. ○——○ normal BME control, ●——● normal BME + bleomycin 0·1 μg/ml, □——□ BME without $Ca^{++}$ and $Mg^{++}$, ▲——▲ BME without $Ca^{++}$ and $Mg^{++}$ + bleomycin 0·1 μg/ml. M.C.—time of medium change.

required more than 200 times as much bleomycin (Fig. 8) and 100 times as much phleomycin to inhibit cell proliferation to the same extent as HeLa S-3 under identical conditions.

*Effect of bleomycin on macromolecular synthesis in HeLa cells*

Since bleomycin exerts its effects on suspension cultures more rapidly than on monolayer cultures, the former were employed for studies of the uptake of radioisotopically labelled thymidine and leucine by HeLa cells exposed to bleomycin at 1 and 5 μg/ml. To emphasize any impairment of DNA synthesis, cultures were synchronized before treatment with amethopterin for 16 hours. When the block was released with thymidine, bleomycin and the radioactive precursors were added. Protein synthesis was unaffected by either 1 or 5 μg/ml bleomycin over 24 hours (see Table I). DNA synthesis was significantly depressed (20–35%) by the end of the synchronous S phase (7–8 hours) with 1 μg/ml bleomycin, and by over 50% with 5 μg/ml bleomycin (Table I). The plateau developing after 7 or 8 hours of [$^3$H]-thymidine incorporation into DNA in bleomycin treated cultures is evidence of the cells failing to progress through their cycle. None of the groups exposed to bleomycin showed an increase in cell number, whereas controls increased by 1·8 to 1·9 fold in 24 hours. In a similar experiment, the rate of protein synthesis was found to be unaltered in synchronized cultures treated with 1 μg/ml bleomycin compared with the controls given 1 hour pulses of [$^3$H]-leucine at

FIG. 8.—Growth of BHK 21/C13/DWS-3 cells in suspension culture in the presence of bleomycin $A_2$. ○——○ control, ●——● 0·1 μg/ml, □——□ 1·0 μg/ml, ☆——☆ 5·0 μg/ml.

intervals up to 10 hours after release from an amethopterin block.

In Table II the rate of RNA synthesis is shown for synchronized and non-synchronized HeLa cultures treated with 1 μg/ml bleomycin as measured by 30 min pulses with labelled cytidine and uridine. No suppression was observed over 24 hours.

Amethopterin blocked cells exposed to bleomycin (1 μg/ml) for the last 4 hours before reversal with thymidine were capable of initiating DNA synthesis to the same extent as controls. Removal of bleomycin during the S phase in these cultures suppressed DNA synthesis to a similar degree to that in which bleomycin was left in the medium. A similar effect was noted by Suzuki *et al.* (1968). The results are interpreted as an irreversible interaction of the antibiotic with DNA and suggest that non-replicating DNA interacts with bleomycin to the same extent as replicating.

TABLE I.—*Effect of Bleomycin (1 and 5 μg/ml) on DNA and Protein Synthesis in Synchronized HeLa S-3 Cells*

| Precursor | Treatment | Average d/min × $10^3$ at hours after reversal (% relative to control) 1 hour | 2 hours | 4 hours | 8 hours | 24 hours |
|---|---|---|---|---|---|---|
| [$^3$H]-Td R | Control | 43·6 | 139·0 | 373·0 | 647·0 | 914·0 |
| | Bleomycin 1 μg/ml | 39·5 (90%) | — | 342·0 (92%) | 421·0 (65%) | 421·0 (46%) |
| | Bleomycin 5 μg/ml | 34·0 (77%) | 108·0 (78%) | 214·0 (57%) | 281·0 (43%) | — |
| [$^{14}$C]-leucine | Control | 3·8 | 6·6 | 12·8 | 24·5 | 66·2 |
| | Bleomycin 1 μg/ml | 3·4 (89%) | — | 15·1 (118%) | 19·4 (80%) | 54·4 (82%) |
| | Bleomycin 5 μg/ml | 3·6 (94%) | 8·8 (135%) | 13·4 (105%) | 22·1 (90%) | 60·4 (91%) |

Amethopterin-synchronized HeLa S-3 cultures were reversed with thymidine which included 0·1 μc/ml thymidine-6-$^3$H, and also supplied with *l*-leucine-1-$^{14}$C at 0·05 μc/ml. Bleomycin or saline was added at this time. At intervals thereafter the accumulated radioactivity incorporated in the acid insoluble fractions was estimated by simultaneous $^3$H and $^{14}$C counting.

TABLE II.—*Incorporation of RNA Precursors into Synchronized HeLa S-3 Cells in the Presence of Bleomycin (1 μg/ml)*

| Precursor | Treatment | Relative percentage of control incorporation of radioisotope after reversal with thymidine (hours) 0·5–1·0 | 1·5–2·0 | 3·5–4·0 | 7·5–8·0 | 23·5–24·0 |
|---|---|---|---|---|---|---|
| *Synchronized cultures* | | | | | | |
| [$^3$H]-cytidine | Bleomycin 1 μg/ml | 112 | 95 | 98 | 103 | 123 |
| [$^3$H]-uridine | Bleomycin 1 μg/ml | 70 | 92 | 112 | 103 | — |
| *Non-synchronized cultures* | | | | | | |
| [$^3$H]-cytidine | Bleomycin 1 μg/ml | 95 | 102 | 121 | 127 | 100 |

Details of experimental design as given in Table 1 except that these cultures received 30 min pulses of tritiated uridine or cytidine

*Colony forming ability of cells after exposure to bleomycin*

Suspension cultured HeLa S-3 cells in exponential growth phase were exposed for periods of up to 24 hours to 1 μg/ml bleomycin $A_2$. Samples were removed at regular intervals, diluted to 100 cells/ml, and 5 ml dispersed into 60 mm petri dishes containing bleomycin-free conditioned monolayer culture medium. The dishes were incubated at 37°C for 8 days. Colonies were counted after 4% neutral buffered formaldehyde fixation and methylene blue staining. Cell viability was not completely lost but within about 2 hours only about 5% survived and at 4 hours viability was negligible (Fig. 9). Whether this represents a short phase of resistance as found with phleomycin (Djordjevic and Kim, 1967) is not known.

When cells were plated out in the same manner as described above from untreated HeLa S-3 suspension cultures into petri dishes containing media with 1 μg/ml bleomycin, some cells persisted for up to one week. Continued incubation of the dishes for 1 month did not result in the appearance of bleomycin resistant colonies. Similarly, colonies never formed in dishes returned to bleomycin free medium for 3–4 weeks after exposure to 1 μg/ml bleomycin for 1 or 2 days.

*Time of action of bleomycin during the cell cycle*

Since bleomycin and phleomycin both have significant inhibitory effects on DNA synthesis, they will inhibit cells from entering mitosis which have not completed replication of the genome, *i.e.* the

FIG. 9.—Clonability of HeLa S-3 suspension cultured cells exposed to 1 μg/ml bleomycin. ●——● Colonies counted only if at least 32 cells (5 divisions) in number, ●– – –● colonies of 8 cells or more.

cells will not enter $G_2$. However, the following experiments demonstrate that (i) bleomycin is a highly effective anti-mitotic agent in the $G_2$ phase, (ii) mitotic cells themselves are not blocked by the antibiotic and (iii) bleomycin can inhibit entry of cells into mitosis at levels which have no quantitatively detectable effect on DNA synthesis.

Synchronized HeLa cells were allowed to synthesize DNA for 8 hours after release from an amethopterin block, *i.e.* approximately the duration of S phase. Colcemid was then added with or without bleomycin. A control given 2 mmol/l hydroxyurea and colcemid was also included. The percentages of arrested metaphases were counted 6 hours later. Addition of hydroxyurea precluded the possibility of cells still in S phase at the time of treatment from reaching mitosis. The $G_2$ population, which is unaffected by hydroxyurea, progressed into mitosis and was arrested by colcemid (Wheatley, 1972). At 6 hours (*i.e.* 14 hours after thymidine reversal) metaphases had reached 26% in the hydroxyurea/colcemid control and 62% in the free-running colcemid control. In contrast, cultures given bleomycin showed a marked inhibition of the accumulation of metaphases which was considerably greater than that caused by the hydroxyurea block, only 9% cells having reached mitosis. It was deduced that 1 μg/ml bleomycin in suspension cultured HeLa cells exerts its maximal anti-mitotic effect in 25–30 min, $G_2$ being 2·5–2·8 hours in duration in this experiment. In similar experiments, the addition of bleomycin or phleomycin together with hydroxyurea to cultures gave identical results to the addition of antibiotic alone. The speed of action of bleomycin is borne out by prophase counts in synchronously growing HeLa cell cultures falling to less than 10% of control values between 30 and 60 min after treatment with bleomycin at 1 μg/ml (Fig. 10a). In this experiment colcemid was also added and the inhibition of metaphase accumulation is clearly shown (Fig. 10b).

FIG. 10.—HeLa S-3 cells in suspension culture synchronized to enter S phase at 0 hour were treated with bleomycin 1 $\mu$g/ml at 8 hours. In 10a (top) prophase index is compared in a treated culture (○- - -○) and an untreated control (●——●). In 10b, the accumulation of metaphases is shown when colcemid was added together with bleomycin 1 $\mu$g/ml (○——○) and on its own (●——●).

Unsynchronized HeLa cell cultures were treated with bleomycin and hydroxyurea to avoid the possibility that the synchronization itself affected the sensitivity of cells to the antibiotic. Bleomycin at 0·5, 1·0 and 10 $\mu$g/ml was added to logarithmically growing HeLa cell suspensions with 2 mmol/l hydroxyurea. Untreated controls maintained a mitotic index of about 4% (Fig. 11). The index in cultures treated with 2 mmol/l hydroxyurea fell to about half in 3 hours and was very low from 4 hours onwards. The area under this curve represents the flow of $G_2$ cells into mitosis (Wheatley, 1972). Bleomycin treatments shifted the curve closer to the ordinate showing that a large part of the $G_2$ cohort had been prevented from progressing into mitosis. Allowing time for cells already in mitosis to move into interphase (30–60 min), the delay before a completely inhibitory

FIG. 11.—Effect of bleomycin on the progression of suspension cultured HeLa S-3 cells into mitosis in asynchronous cultures. ×——× control, ▲——▲ 2 mmol/l hydroxyurea, △——△ 1 μg/ml bleomycin, ◆——◆ 5 μg/ml, ○- - -○ 10 μg/ml.

TABLE III.—*Effect of Bleomycin (10–100 μg/ml) on Prophases in HeLa S-3 Suspension Cultures*

| Bleomycin μg/ml | Percentage prophases at time after treatment (min) | | | | | |
|---|---|---|---|---|---|---|
| | 10 | 20 | 30 | 45 | 60 | 90 |
| Control | 0·26 | 0·28 | 0·35 | 0·30 | 0·30 | 0·26 |
| 10 μg/ml | 0·12 | 0·15 | 0·10 | 0·12 | 0·00 | 0·02 |
| 20 μg/ml | 0·02 | 0·00 | 0·07 | 0·05 | 0·12 | 0·07 |
| 50 μg/ml | 0·00 | 0·00 | 0·00 | 0·00 | 0·00 | 0·00 |
| 100 μg/ml | 0·05 | 0·00 | 0·00 | 0·00 | 0·00 | 0·00 |

Prophases were counted in duplicate cultures for each time point, not less than 2000 cells being included in each individual count.

TABLE IV.—*DNA Synthesis in Synchronized HeLa S-3 Cultures Treated with Phleomycin and Bleomycin (0·1 μg/ml) and the Ability of Cells to Reach Mitosis*

| Treatment | [$^3$H]-Thymidine incorporation ct/min × $10^3$/μg protein (percentage of control) at stated hours after reversal | | | | Relative increase in cell number as percentage of control at 13·5 hours |
|---|---|---|---|---|---|
| | 2 hours | 4 hours | 7 hours | 13·5 hours | |
| Control | 58·4 (100) | 133·0 (100) | 173·2 (100) | 213·0 (100) | 100 |
| Bleomycin 0·1 μg/ml | 60·7 (104) | 128·8 (97) | 176·3 (102) | 227·0 (107) | 66 |
| Phleomycin 0·1 μg/ml | 63·0 (108) | 128·6 (97) | 180·8 (104) | 222·0 (105) | 56 |

Thymidine-6-$^3$H (0·1 μCi/ml, 4 mCi/mmol) incorporation measured in duplicate suspension cultures of HeLa S-3 cells after reversal from amethopterin block.

action of 10 $\mu$g/ml bleomycin was observed must be considerably less than 30 min. Using high dose levels of bleomycin (10–100 $\mu$g/ml) an inhibitory effect on progression of cells into prophase was seen within minutes of treatment (Table III). Bleomycin has no inhibitory effect on the progression of mitotic cells through division at dose levels well above those required to arrest entry into mitosis (5–10 $\mu$g/ml) (Fig. 12). Finally, it was

FIG. 12.—Mitotic HeLa cells collected by mechanical dislodgement were immediately exposed (0 hour) to bleomycin 5 $\mu$g/ml (○——○) or medium without the antibiotic (●——●).

demonstrated that very low dose levels of bleomycin and phleomycin can significantly suppress division in suspension cultured HeLa cells without quantitatively affecting the amount of DNA synthesis within the cells (Table IV).

### DISCUSSION

While the effects of bleomycin on the growth kinetics of HeLa cells in monolayer culture reported here are generally in agreement with the findings of others (Ichikawa *et al.*, 1967; Kunimoto *et al.*, 1967; Suzuki *et al.*, 1968; Tanaka *et al.*, 1963b), the most important difference is the demonstration of a far greater sensitivity in suspension culture, which also applies to phleomycin. This has been largely attributable to the absence of divalent ions. Permeability of cells to the antibiotics could be considerably affected by the presence of $Ca^{++}$ and $Mg^{++}$ although Pietsch *et al.* (1969) maintain that phleomycin is freely diffusible across biological membranes. This possibility is being investigated for bleomycin. Sensitivity was also found to vary with cell type, in general epithelial cells being more susceptible than fibroblasts. Indeed, HeLa cells in suspension culture are two orders of magnitude more sensitive than the suspension adapted sub-strain of the BHK 21/C13 cell line (DWS-3) grown under identical conditions. This contrasts with the report of Terasima and Umezawa (1970) that mouse L-strain fibroblasts of the L5 sub-strain are considerably more sensitive than HeLa cells. However, comparisons of results from different laboratories where different cells, culture methods and batch preparations of antibiotic have been used, are unlikely to be particularly reliable. In the present study, an attempt has been made to standardize the culture medium before testing with the same preparation of antibiotic; differences in sensitivity are probably due to inherent properties of the cells themselves.

The ability of a particular cell type to concentrate bleomycin once it has gained access to the cell may also play a part in differential sensitivity. There is some evidence from *in vivo* studies that epithelial cells (*e.g.* the basal layer of the epidermis) concentrate [$^3$H]-bleomycin (Umezawa *et al.*, 1968*a*). Another possibility is that certain cells might inactivate bleomycin by releasing proteolytic enzymes just as *in vitro* treatment of bleomycin with pronase can inactivate it (Haidle, 1971). In this laboratory we have passed medium containing bleomycin at critical inhibitory concentrations from one culture to another over 5–6 days without detecting loss of inhibitory action. An alternative is that cell sensitivity is inversely related to antibiotic activation within the cell (Müller *et al.*, 1972).

Sensitivity differences could be related to the access of bleomycin to the DNA, its degree of binding and the amount of excision damage it can produce. These points are dealt with elsewhere (Müller *et al.*, 1972) and the possibility need only be raised here of $Ca^{++}$ or $Mg^{++}$ affecting the interaction of bleomycin with the target molecules rather than some earlier sequence in the chain of events. The value of bleomycin in chemotherapy lies chiefly in its selectivity of action on certain tissue elements. The underlying causes of the differences in sensitivity discussed here warrant further investigation since they may help to elucidate the mechanism of action and to improve the design of selective chemotherapeutic agents.

Bleomycin and phleomycin have a particularly powerful inhibitory action on post-S phase ($G_2$) cells. Their action on suspension cultured HeLa cells is very rapid even at low concentrations. Virtually no delay in disappearance of prophases was seen with high dose levels of bleomycin (Table III) which suggests that there is no completely resistant phase at the end of $G_2$. The antibiotics both act over the entire $G_2$ period, which agrees with the finding of Djordjevic and Kim (1967). In plant cells, bleomycin and phleomycin are also effective inhibitors throughout $G_2$ (Hotta and Stern, 1969).

The suppression of DNA synthesis by the antibiotics potentiates their antimitotic activity. We have found that very low concentrations of bleomycin (0·1 $\mu$g/ml or less) exert some antimitotic action on HeLa S-3 suspension cells in the absence of quantitatively detectable suppression of DNA synthesis, suggesting that $G_2$ events are considerably more sensitive than DNA replication. The interference of the antibiotics with DNA replication is becoming clearer from recent studies (Suzuki *et al.*, 1969, 1970; Terasima, Yasukawa and Umezawa, 1970; Haidle, 1971; Müller *et al.*, 1972). It has been shown that bleomycin causes strand scission of DNA, but, unlike irradiation-induced mitotic delay, it is suspected that damage cannot be as easily repaired and cells are permanently held in $G_2$. The cleavage of DNA molecules by bleomycin must occur in the post-replication phase if the mechanism of inhibiting cell entry into mitosis is radiomimetic in nature. We are presently attempting to correlate the degree of strand scission with the sensitivity of different cell lines, and the same cell lines under different conditions, to bleomycin.

It is of particular interest that transcriptional processes are quantitatively unaffected by the interaction of the antibiotics with DNA until long after mitotic activity has ceased and DNA replication suppressed. Little is known of the site of interaction between bleomycin and DNA although the site of interaction of phleomycin with DNA is different from that of actinomycin D which does interfere with transcription (Pietsch, 1969).

The use of the antibiotics in cell cycle analyses to collect and hold cells in $G_2$ without quantitatively altering transcriptional and translational processes should make it possible to compare genetic expression in $G_2$ with that at other stages of the cycle. Studies of this nature are in progress (Wheatley, in preparation). Qualitative and quantitative differences in proteins synthesized by $G_2$ cells compared with cells at other stages of the cell cycle may exist (Kolodny and Gross, 1969). It is hoped that bleomycin and phleomycin will help to elucidate the significance of the $G_2$ phase of the cell cycle and the trigger mechanism for mitosis. Their effectiveness at low concentrations in HeLa S-3 cells, the irreversibility of their action and the lack of early interference with RNA and protein synthesis suggest that a very specific intracellular target is involved.

Much of this was carried out during tenure of a Medical Research Council Travelling Fellowship at the McArdle Laboratory for Cancer Research, University of Wisconsin, Madison, Wisconsin 53706, U.S.A. (DNW). It was also supported by the Cancer Research Campaign. Technical assistance was provided by Mrs Sandra Remmer and Mrs Marget Inglis.

### REFERENCES

CERIOTTI, G. (1955) Determination of Nucleic Acids in Animal Tissues. *J. biol. Chem.*, **214**, 59.

CLINICAL SCREENING CO-OPERATIVE GROUP of E.O.R.T.C. (1970) Study of the Clinical Efficiency of Bleomycin in Human Cancer. *Br. med. J.*, ii, 643.

DJORDJEVIC, B. & KIM, J. H. (1967) Lethal Effects of Phleomycin in Different Stages of the Division Cycle of HeLa cells. *Cancer Res.*, **27**, 2255.

HAIDLE, C. W. (1971) Fragmentation of Deoxyribonucleic acid by Bleomycin. *Mol. Pharmacol.*, **7**, 645.

HOTTA, Y. & STERN, H. (1969) The Action of Phleomycin on Meiotic Cells. *Cancer Res.*, **29**, 1699.

ICHIKAWA, T., MAEDA, A., YAMAMAOTO, K., TSUBOSAKI, M., KAIHARA, T., SAKAMOTO, M. & UMEZAWA, H. (1967) Biological Studies on Bleomycin A. *J. Antibiot.*, Ser. A., **20**, 149.

ISHIZUKA, M., TAKAYAMA, H., TAKEUCHI, T. & UMEZAWA, H. (1967) Activity and Toxicity of Bleomycin. *J. Antibiot.*, Ser. A, **20**, 15.

KAJIWARA, K., KIM, U. H. & MUELLER, G. C. (1966) Phleomycin, an Inhibitor of Replication of HeLa Cells. *Cancer Res.*, **26**, 233.

KISSANE, J. M. & ROBBINS, E. (1959) The Fluorimetric Measurement of Deoxyribonucleic Acid in Animal Tissues, with Special Reference to the Central Nervous System. *J. biol. Chem.*, **233**, 184.

KOLODNY, G. M. & GROSS, P. R. (1969) Changes in Patterns of Protein Synthesis during the Mammalian Cell Cycle. *Expl Cell Res.*, **56**, 117.

KUNIMOTO, T., HORI, M. & UMEZAWA, H. (1967) Modes of Action of Phleomycin, Bleomycin and Formycin on HeLa S-3 Cells in Synchronised Culture. *J. Antibiot.*, Ser. A, **20**, 277.

MUELLER, G. C., KAJIWARA, K., STUBBLEFIELD, E. & REUCKERT, R. R. (1962) Molecular Events in the Reproduction of Animal Cells. I. The Effect of Puromycin on the Duplication of DNA. *Cancer Res.*, **22**, 1084.

MUELLER, G. C. (1971) Biochemical Perspectives of the $G_1$ and S Intervals in the Replication Cycle of Animal Cells: A Study in the Control Cell Growth. In *The Cell Cycle and Cancer.* Ed. Baserga. New York: Marcel Dekker. p. 282.

MÜLLER, W. E. G., YAMAZAKI, Z-I., BRETER, H.-J. & ZAHN, R. K. (1972) Action of Bleomycin on DNA and RNA. *Eur. J. Biochem.*, **31**, 518.

OGAWA, K. & ONOÉ, T. (1969) Nuclear Changes Produced by Bleomycin in the 3-Methylcholanthrene-Induced Mouse Epidermal Carcinoma Cells. *Gann*, **60**, 503.

OYAMA, V. I. & EAGLE, H. (1956) Measurement of Cell Growth in Tissue Culture with a Phenol Reagent (Folin-Ciocalteau). *Proc. Soc. exp. Biol. Med.*, **91**, 305.

PIETSCH, P. (1969) Structural Events in DNA in Transcription and Replication: The Influence of Histones on *in vitro* Reactions of Actinomycin D and Phleomycin-909. *Cytobios.*, **4**, 375.

PIETSCH, P. & CLAPPER, G. (1969) Receptivity of DNA to Phleomycin. *Cytobios.*, **2**, 145.

PIETSCH, P., CORBETT, C., BRIDEN, D. W. & JEWETT, G. (1969) Diffusability of Phleomycin by means of Neutron Activation Analysis. *Physiol. Chem. & Phys.*, **1**, 232.

PIETSCH, P. & GARRETT, H. (1968) The Primary Site of Reaction in the *in vitro* Complex of Phleomycin in DNA. *Nature, Lond.*, **219**, 488.

SCHINDLER, R. (1963) Biochemical Studies on the Division Cycle of Mammalian Cells: Evidence for the Pre-Mitotic Period. *Biochem. Pharmac.*, **12**, 533.

SHASTRI, S., SLAYTON, R. E., WOLTER, J., PERLIA, C. P. & TAYLOR, S. G. III. (1971) Clinical Study with Bleomycin. *Cancer, N.Y.*, **28**, 1142.

SUZUKI, H., NAGAI, K., AKUTSU, E., YAMAKI, H., TANAKA, N. & UMEZAWA, H. (1970) On the Mechanism of Action of Bleomycin. Strand Scission of DNA Caused by Bleomycin and its Binding to DNA *in vitro* *J. Antibiot.* Ser. A,

SUZUKI, H., NAGAI, K., YAMAKI, H., TANAKA, N. & UMEZAWA, H. (1968) Mechanism of Action of Bleomycin. Studies with the Growing Culture of Bacterial and Tumour Cells. *J. Antibiot.*, Ser. A, **21**, 379.

SUZUKI, H., NAGAI, K., YAMAKI, H., TANAKA, N. & UMEZAWA, H. (1969) On the Mechanism of Action of Bleomycin: Scission of DNA strands *in vitro* and *in vivo*. *J. Antibiot.*, Ser. A, **22**, 446.

TANAKA, N., YAMAGUCHI, H. & UMEZAWA, H. (1963*a*) Mechanism of Action of Phleomycin, A Tumour-Inhibitory Antibiotic. *Biochem. biophys. Res. Commun.*, **10**, 171.

TANAKA, N., YAMAGUCHI, H. & UMEZAWA, H. Tumour-inhibitory Antibiotic. *Biochem. biophys.* (1963*b*) Mechanism of Action of Phleomycin. I. Selective Inhibition of the DNA Synthesis in *E. coli* and in HeLa Cells. *J. Antibiot.*, Ser. A, **16**, 86.

TERASIMA T. & UMEZAWA, H. (1970) Lethal Effect of Bleomycin on Cultured Mammalian Cells. *J. Antibiot.* Ser. A, **23**, 300.

TERASIMA, T., YASUKAWA, M. & UMEZAWA, H. (1970) Breaks and Rejoining of DNA in Cultured Mammalian Cells Treated with Bleomycin. *Gann*, **61**, 513.

UMEZAWA, H., ISHIZUKA, M., HORI, S., CHIMURA, H., TAKEUCHI, T. & KOMAI, T. (1968*a*) The Distribution of $^3$H-Bleomycin in Mouse Tissue. *J. Antibiot.*, Ser. A, **21**, 638.

UMEZAWA, H., ISHIZUKA, M., KIMURA, K., IWANAGA, J. & TAKEUCHI, T. (1968*b*) Biological Studies on Individual Bleomycins. *J. Antibiot.*, Ser. A, **21**, 592.

UMEZAWA, H., MAEDA, K., TAKEUCHI, T. & OKAMI, Y. (1966) New Antibiotics, Bleomycin A and B. *J. Antibiot.*, Ser. A, **19**, 200.

WHEATLEY, D. N. (1972) Action of Adriamycin on HeLa Cells. Evidence of a $G_2$ Inhibition. In *International Symposium on Adriamycin.* Ed. Carter *et al.* Berlin: Springer Verlag. p. 47.